# Characterization of seahorse (*Hippocampus comes* L.) extracts originating from culture and nature in Pesawaran, Lampung, Indonesia

**DOI:** 10.5455/javar.2022.i630

**Published:** 2022-11-19

**Authors:** Trisnawati Mundijo, Franciscus Dhyanagiri Suyatna, Agung Eru Wibowo, Agus Supriyono, Yurnadi Hanafi Midoen

**Affiliations:** 1 Doctoral Programme Biomedical Science, Faculty of Medicine, Universitas Indonesia, Jakarta, Indonesia; 2 Department of Medical Biology, Faculty of Medicine, Universitas Muhammadiyah Palembang, Palembang, Indonesia; 3 Department of Pharmacology and Therapeutics, Faculty of Medicine, Universitas Indonesia, Jakarta, Indonesia; 4 Research Center for Pharmaceutical Ingredients and Traditional Medicine, National Research and Innovation Agency, Tangerang, Indonesia; 5 Department of Medical Biology, Faculty of Medicine, Universitas Indonesia, Jakarta, Indonesia

**Keywords:** *Hippocampus comes* L., amino acid, steroid, cultured, nature

## Abstract

**Objective:**

Indonesia is an archipelagic country with a mega biodiversity, among others, in the marine area. Seahorses (*Hippocampus* spp.) are a marine fish known to have biocompounds used in traditional medicine “Jamu,” such as *Hippocampus comes* L. (HCL). The present study aims to analyze and compare the chemical contents of cultured seahorse (CS) and natural seahorse (NS) extracts.

**Materials and Methods:**

The CS and NS were identified morphometrically. After freeze-drying, the seahorses were ground into powder with a grinder. The seahorse powder was extracted with ethanol and a water solvent. The extract contained biocompounds, proximate, amino acids, and steroids with high-performance liquid chromatography.

**Results:**

The study found unique characteristics of HCL. The highest yield was obtained in NS using a water solvent (18.6%). The biocompounds in seahorses consist of alkaloids and triterpenoids. The highest proximate of water content (11.03%) and ash content (42.50%) was found in NS. In addition, other compounds were also detected in CS, such as fat (7.48%) and protein (47.67%). Both of HCL’s different sources had all essential and nonessential amino acids in which the highest concentration were in NS, i.e., L-arginine (56,537.22 mg/kg), L-lysine (17,794.17), glycine (113,649.80 mg/kg), L-proline (47,056.15), and L-alanine (43,451.81). The analysis of the steroid compound of the extract suggested the presence of steroid glycosides.

**Conclusion:**

The highest yield of the seahorse extract with a water solvent is about 18.6% and protein content of 47.67% in CS. The crude extract has alkaloids, triterpenoids, and glycine (113,649.80 mg/kg) in NS with water, suggesting the presence of steroid glycosides.

## Introduction

Seahorse is a marine fish with great potential as a fishing commodity, in the fishing industry, and as a global food [1,2]. There are more than 50 species of seahorses worldwide [3], and 33 species are found in Indonesia [4]. One of the seahorse species in Indonesia is the *Hippocampus* *comes* L. (HCL), which is the most used in traditional medicine because of its medicinal value [5–7]. In Indonesia, HCL is used in traditional medicine as a “Jamu” [4] because it has biocompounds including the aphrodisiac effect, respiratory system, immune system, and antioxidant, antifatigue, and anti-inflammatory properties [8,9]. The bioactive compounds in seahorse extract are steroids, amino acids, protein, taurine, fatty acids, cholesterol, and trace elements [10,11].

To date, research on seahorses in Indonesia commonly uses ethanol as a solvent. Several reports described the extraction with ethanol [5], water [12], ether acetate [13], and methanol [14]. However, no previous research has compared HCL extraction solvents to ethanol and water.

Qian et al. [15] reported the extraction with water, methanol, and ethanol used in *Hippocampus kuda* L. Sanaye et al. [16] also used methanol in the extraction of *Hippocampus kuda L.* [8]. However, methanol is not recommended because it can be toxic for humans [17].

So far, studies are yet to document the findings of HCL analysis from both culture and nature. There was only one study, but it utilized a different species. There is a discrepancy between the existing data and the secondary metabolite level of HCL, which is the most common species in Indonesia. Previous studies in Indonesia by Sari et al. [5] and Safryna [10] revealed that HCL extracted with ethanol solvents contained amino acids and steroids. Using a qualitative test, the study identified the possible presence of steroids. The objective of this study is to quantify the steroids using the high-performance liquid chromatography (HPLC) test. In the present study, we quantify the steroids and other compounds of HCL from different habitats using HPLC with different solvents. This study provides a scientific reason for using HCL to help people’s health.

## Materials and Methods

### Ethical approval

The ethics committee of the Faculty of Medicine, Universitas Indonesia, approved this study with protocol number KET-101/UN2.F1/ETIK/PPM.00.02/2021.

### Morphological identification

Morphological identification was made according to the guidelines for seahorse identification [18], obtained from meristic measurements and the unique morphology [10,11].

### Samples

Male and female HCL seahorses were obtained from culture and nature. The cultured seahorse (CS) was collected from the Marine Cultivation Fisheries Center, Marine and Fisheries Department, Lampung, Indonesia. The CS was obtained from Juwana, a tampon tank with aeration. During the juvenile phase, the CS was fed zooplankton like *copepods*, *Nauplii*, and* Artemia* sp. Shrimps, *Artemia* sp., and *Nauplii* were given after the next development phase, when the size of CS was more than 2 cm. The feedings were done three times a day. The minimal length of an adult seahorse used in the study was 12 cm. Only adult seahorses are commonly used for traditional medicine [19].

The natural seahorse (NS) was collected from the fishers by Karya Usaha Bersama, Karya Laut, Pesawaran, and Lampung, with supervision from the Marine Cultivation Fisheries Center, Marine and Fisheries Department, Lampung, Indonesia.

### Sample collection

The seahorses were stabilized with fish stabilizer (3 ml in 12 l of water), washed with water, and frozen with a freeze dryer (Heto FD4 Diagnostic) for 48 h at −45°C.

The freeze-dried samples were weighed and crushed into powder using a Retsch grinder. The seahorse powder was analyzed for proximate, water content, ash, fat, protein, and carbohydrates, according to the National Standardization Agency, Indonesia [20].

The extraction was done with 5 gm of powder from CS and NS using ethanol and water as the solvent to find the yield*,* crude extract compounds, amino acids, and steroids. The results for the yield were divided by the total weight (%) [10].

Identifying alkaloids, steroids, triterpenoids, saponin, flavonoids, and tannin was conducted using reagents Dragendrof, Mayer, and Wagner. The steroid and triterpenoid were analyzed using the Liebermann–Buchard method. Saponin and tannin contents were analyzed using magnesium (Mg) and flavonoids were analyzed using chloride acid (HCl) and amyl alcohol [5]. Detection and quantification of amino acids were conducted using the HPLC detector fluorescence and HPLC PDA. Steroids were analyzed using the UV-VIS C-18 column [5,10,21,22].

### Ethanol extraction

Ethanol (96%) was used in the maceration of 5 gm seahorse powder in 20 ml of solvent (w/v) [5]. Maceration was done for 24 h and filtered with Whatman 42. Next, the macerated HCL was processed using the rotary evaporator Heidolph, Buchi Vacuum Pump V-700, 20°C–25°C.

### Water extraction

Five grams of macerated seahorse powder were extracted with 50 ml of phosphate buffer for 24 h and stirred at 500 rpm for 2 h [15]. The mixture was centrifuged [11], and the supernatant was frozen for 48 h at −45°C and stored at −80°C [21].

## Results

### Morphology of a seahorse

**Figure 1.**
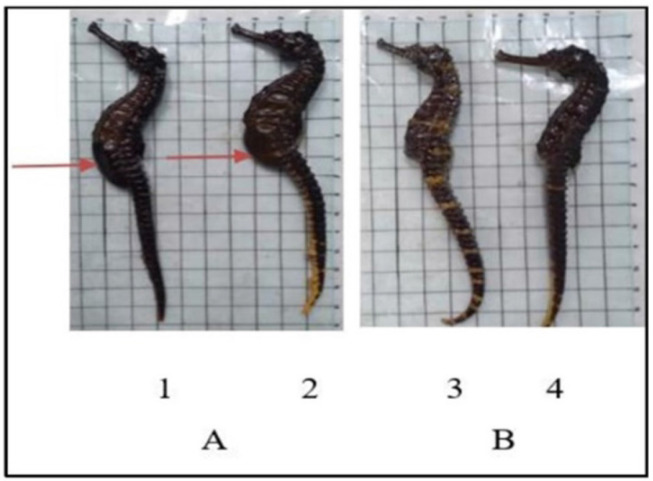
HCL from a private collection. A: Male; B: Female; 1 and 4: NS; and 2 and 3: CS. Arrows: Brood pouch in male seahorses.

The HCL from Pesawaran Lampung, has a unique morphology, as shown in Figure 1.

### Proximate tests

**Figure 2.**
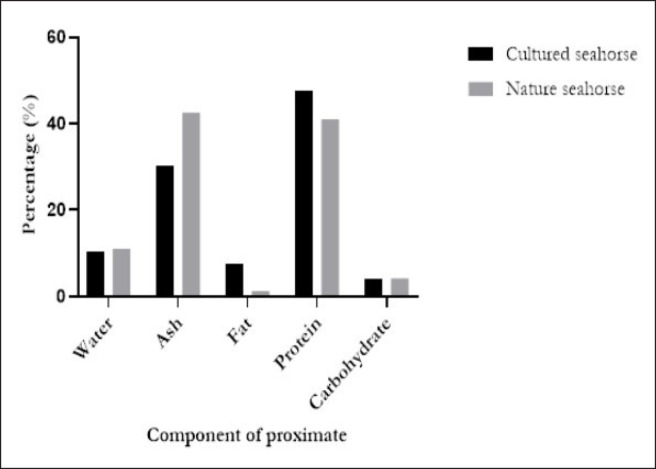
The proximate test of HCL. Fat content (7.48%) and protein content (47.67%) were highest in CS; on the other hand, water content (11.03%), ash content (42.50%), and carbohydrate (4.18%) were mostly high in NS.

The proximate analysis is shown in Figure 2, of which water (11.03%) and ash content (42.50%) were the highest constituents in NS. Fat (7.48%) and protein content (47.67%) were the highest in CS.

### Extraction

The extraction details are shown in Table 1. The results show that the highest yield in water solvent for CS and NS extractions was, respectively, 15.4% and 18.6%. The alkaloid and triterpenoid content of the extract were also high.

Essential and nonessential amino acids were detected in both seahorses (Table 2), in which NS had the highest contents of L-arginine (56,537.22 mg/kg), L-lysine (17,794.17), glycine (113,649.80 mg/kg), L-proline (47,056.15), and L-alanine (43,451.81).

The analysis of steroids showed no similar steroids to the standard. The peak chromatographic details are shown in Table 3.

### HPLC for steroid

**Figure 3.**
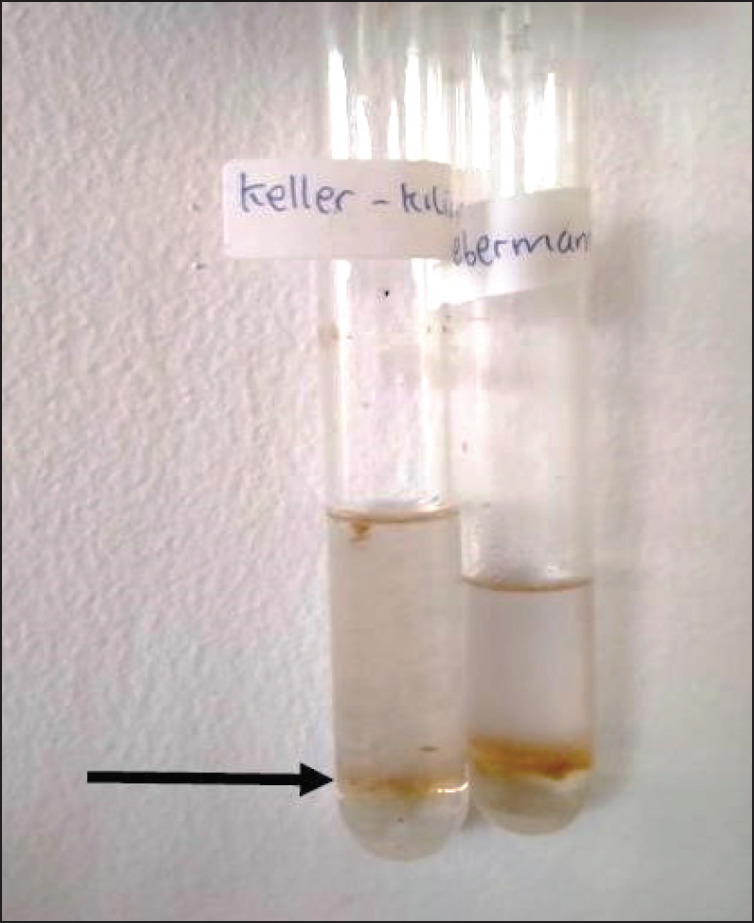
Keller–Killiani test shows a ring (arrow). It clearly shows a positive result with a brown ring, indicating the presence of steroid compounds.

The HPLC and absorbance profile in Table 3 show that the peaks of the analyzed extract were almost as high as the standards. In addition, the retention time of the extract chromatogram appeared earlier than the standard. The NS extract with a water solvent had the best results compared with other extracts as indicated by the profile similarity and maximum absorbance according to electroVolt absorbance, i.e., 750 mAU for ethinyl estradiol, 1.000 mAU for methyl testosterone, and 500 mAU for progesterone. We also confirmed the presence of steroid glycosides with the Keller–Killiani assay (Fig. 3).

## Discussion

In the present study, we used adult HCL from Pesawaran, Lampung, at least 12 cm in length. Similar to previously reported studies, we found a unique morphology in the seahorses with double spine cheeks, patches on the body, and a reddish yellow color on the tail [4,5,9–11,21].

In our study, we used a seahorse, at least 12 cm in length, which is an adult and used for traditional medicine [19]. In addition to that, the Convention on International Trades in Endangered Species (CITES) recommends that the length of a seahorse be at least 10 cm [23] to avoid extinction in wild nature [24].

The novelty in our study is the characterization of the extracts in which we compare the NS and CS with different solvents. We found that the yield from a water solvent was the highest percentage in NS (18.6%). In proximate analysis, fat content (7.48%) and protein content (47.67%) were higher in CS, but the water (11.03%), ash (42.50%), and carbohydrate contents (4.18%) were higher in NS. The protein content, the highest constituent in proximate analysis, was similar to that in previous studies [5,10,25]. Our study found alkaloids and triterpenoids, which are different from other studies conducted in Indonesia [5,10].

**Table 1.** The characterization of the HCL extract.

Parameters	CS		NS	
	Ethanol solvent	Water solvent	Ethanol solvent	Water solvent
Yield (%)	7.0	15.4	11.4	18.6
Biocompounds				
Alkaloid	**+**	**+**	**+**	**+**
Flavonoid	−	−	−	−
Saponin	−	−	−	−
Steroid	−	−	−	−
Triterpenoid	**+**	**+**	**+**	**+**
Tannin	−	−	−	−
Steroids				
Ethinyl estradiol	−	−	−	−
Methyl testosterone	−	−	−	−
Progesterone	−	−	−	−

The NS extract has a high yield with a water solvent (18.6%); only has alkaloids and triterpenoids; and there is none of the same steroids with the standards.

(+): Identified, (−): Not identified.

In our study, we found that amino acids L-arginine (56,537.22 mg/kg), L-lysine (17,794.17), glycine (113,649.80 mg/kg), L-proline (47,056.15), and L-alanine (43,451.81) were the highest in NS. These data suggested a favorable condition for the NS to have a better survival capacity in their living environment than CS. The NS must be able to adapt to changes in the marine environment consisting of various climate changes, reclamation, pollution, etc. [2]. This study found that the presence of high amounts of amino acids in HCL may potentially be used for nutraceutical natural products from marine sources. Marine organisms are natural products with structure and bioactivity that are highly different from those on the mainland. This study also increases our interest in exploring drug development opportunities from natural products [23,26].

**Table 2.** Amino acids in seahorses (the levels are presented from the highest to the lowest in NS).

Compound name	Level of amino acids (mg/kg)	
	CS	NS
Essential L-Arginine L-Phenylalanine L-Threonine L-Leucine L-Valine L-Lysine L-Histidine L-Isoleucine L-Methionine L-Tryptophane Nonessential Glycine L-Proline L-Glutamate Acid L-Alanine L-Aspartate Acid L-Serine L-Tyrosine L-Sistine	54,743.61 27,695.61 24,034.43 26,880.58 20,007.96 17,355.09 14,368.55 13,671.32 3,645.71 2,281.78 90,909.58 39,633.42 46,796.58 40,175.12 29,895.15 26,273.32 17,523.82 1,161.92	56,537.22 23,213.67 22,491.30 21,904.59 19,287.34 17,794.17 12,725.72 11,335.90 3,357.61 1,300.00 113,649.80 47,056.15 45,812.52 43,451.81 29,559.04 23,958.78 13,003.43 863.57

The HCL seahorse extracts have essential and nonessential amino acids, with the highest levels in L-arginine (56,537.22 mg/kg), L-lysine (17,794.17), glycine (113,649.80 mg/kg), L-proline (47,056.15), and L-alanine (43,451.81) in NS.

We observed that the amino acids in our results differ from those of Sun et al. [21], who reported that the highest level of amino acids came from CS. The difference might be partly due to the studied species and the body tissue of the seahorse used for extraction.

Our study is the first in Indonesia to use the HPLC method to find the steroid in HCL. The chemical components in HCL indicated the presence of steroid glycosides in both HCL and solvents. The analyzed quantitative steroids with liquid chromatography-mass spectrometry (LC-MS) showed that the four compounds with Mw of 240, 619, 717, and 755 were not ethinyl estradiol, methyltestosterone, or progesterone, which were used as standards. Indeed, we detected the compounds as peaks in the HPLC with an earlier appearance of retention time and gave positive results with Keller–Killiani reactions. The positive results with the Keller–Killiani reaction suggested the presence of steroid glycosides in HCL with sugar (glycoses) and nonsugar (aglycones) side chains. Aglycones are commonly a secondary metabolite from steroids, alkaloids, and saponin compounds that can influence biological activity. During extreme conditions, some fish can produce this metabolite, such as diosgenin and aglycone compounds. Steroid glycosides in marine organisms have a different structure in comparison with terrestrial organisms, which contain steroid aglycones and carbohydrates. Exploration of steroid glycosides from HCL and marine organisms is needed in the future, considering that 70% of the world is covered by the sea [27–30].

Another study found steroids and cholesterol to be from different species of seahorses. Other authors found oxacycloheptadecan-2-one, chrysophanol, cholesteryl benzoate, eicosapentaenoic acid, and docosahexaenoic acid in *Hippocampus erectus* [14]. Wu et al. [31] found cholest-4-en-3-one, 3-β-hydroxycholest-5-en-7-one, cholest-5-ene-3β, 7β-diol, cholest-5-ene-3β, 7α-diol in *Hippocampus* *trimaculatus*.

**Table 3.** Data on HPLC with retention time and wavelength values of standard compounds and compounds in seahorse extracts.

Standard compounds/extract	Retention time	UV absorbance (nm)	mAU
Ethinyl estradiol	4.100	248.79; 279.98; 356.13	300
Methyl testosterone	3.500	208.13; 240.93; 282.68; 374.48; 486.99; 521.75; 654.97; 749.18	300
Progesterone	7.408	202.78; 242.29; 361.05	50
Ethanol extract of CS	3.005 2.705 2.555	239.4; 347.76 485.83; 550.37; 577.35; 666.93; 738.28. -	110 300 200
Water extract of CS	2.549 2.693 2.566	248.84; 275.66 249.13; 273.64; 485.76; 514.11; 581.14; 631.80; 665.5; 756.46 248.61; 274.86	350 600 350
Ethanol extract of NS	2.510 2.672 3.057	240.74; 251.75; 350.73 193.36; 240.2; 424.99; 485.39; 581.03; 663.85 239.65; 250.69; 355.01	70 150 50
Water extract of NS	2.472 2.707 2.580	252.81; 274.91 195.51; 252.39; 467.55; 581.54; 631.77; 655.6; 748.03 253.64; 274.48	750 1,000 500

From these data, the results of the study consisting of yield value, proximate content, biocompounds, amino acids, and steroid glycosides conclude that NS with a water solvent has more optimal results than CS with an ethanol solvent. Therefore, HCL seahorses in nature with a water solvent are feasible to explore and to investigate its value in traditional medicine.

## Conclusion

The HCL seahorse extracts from Pesawaran, Lampung, Indonesia, has a high yield in CS with a water solvent of about 18.6%, protein of 47.67%, and only identified alkaloids and triterpenoids. The most elevated amino acids are shown in NS with a water solvent and an indicated steroid glycoside. The results of this study will be helpful in further reviewing data on seahorses, especially in Indonesia, which potentially can be used as a natural product for medicinal use.

## List of abbreviations

HCL. *Hippocampus comes* L.; CS, Cultured seahorse; NS, Nature seahorse; HPLC, High-performance liquid chromatography; LC-MS, Liquid Chromatography-Mass Spectrometry; UV, Ultraviolet; NSA, National Standardization Agency; CITES, Convention on International Trades in Endangered Species.
